# Novel implant design of the proximal interphalangeal joint using an optimized rolling contact joint mechanism

**DOI:** 10.1186/s13018-019-1234-6

**Published:** 2019-07-12

**Authors:** Seok Woo Hong, Junsuk Yoon, Yong-Jae Kim, Hyun Sik Gong

**Affiliations:** 1 Department of Orthopedic Surgery, Kangbuk Samsung Hospital, Sungkyunkwan University School of Medicine, 29, Saemunan-ro, Jongno-gu, Seoul, 03181 Republic of Korea; 2 Department of Electrical Engineering, Korea University of Technology and Education, 1600, ChungJeol-ro, Dongnam-gu, Cheonan-si, Chungcheongnam-do 31253 Republic of Korea; 3 Department of Orthopedic Surgery, Seoul National University Bundang Hospital, Seoul National University College of Medicine, 82, Gumi-ro, 173-beon-gil, Bundang-gu, Seungnam, 13620 Republic of Korea

**Keywords:** Proximal interphalangeal joint implant, Rolling contact joint mechanism, Average center of rotation, Tendon excursion, Constrained optimization

## Abstract

**Background:**

The aims of this study were to propose a novel implant design for the proximal interphalangeal joint (PIPJ) of the hand using a rolling contact joint (RCJ) mechanism and to derive an optimal implant design based on human PIPJ kinematics.

**Methods:**

In total, 10 participants with normal PIPJs were enrolled in this study. True lateral finger radiographs were obtained in 10° increments from 0º (full extension) to 120° flexion of PIPJ. Radiographs were used to determine the average center of rotation, which formed the basis of a mathematical expression of the PIPJ kinematics. The variations in extensor tendon excursions in relation to the range of motion of PIPJ were determined using results from previous cadaveric studies. As the next step, a PIPJ implant design using an RCJ mechanism that was most consistent with the mathematically expressed PIPJ kinematics and tendon excursions was determined using a constrained optimization algorithm.

**Results:**

The final proposed PIPJ implant had a relatively constant center of rotation over the entire PIPJ range of motion among the participants. In addition, the extensor tendon excursions of the proposed implant as applied to the phalangeal bones were similar to those of the human tendon. The proposed PIPJ implant achieved an acceptable position of the RCJ surface on the proximal and middle phalanges, which was derived from the constrained optimization algorithm.

**Conclusions:**

A novel PIPJ implant design using an RCJ mechanism demonstrated acceptable outcomes in terms of PIPJ human kinematics and tendon excursions.

## Introduction

Joint replacement arthroplasty of the proximal interphalangeal joint (PIPJ) can be performed as the salvage procedures when the joint is destroyed due to reasons such as degenerative arthritis, inflammatory arthritis, and post-traumatic arthritis [1]. In particular, post-traumatic arthritis is a well-known complication of articular fracture associated with PIPJ dislocation, and total PIPJ replacement arthroplasty might be one of the best options for reducing the pain and range-of-motion limitation in patients with post-traumatic arthritis due to the PIPJ dislocation [2, 3]. Several studies have been conducted to develop implants used in PIPJ replacement arthroplasty.

Various implant designs for the PIPJ have been proposed in clinical practice. In particular, the following two types of designs have been suggested: the constrained design, that has a hinge structure, and the unconstrained design, that uses surface replacement [4]. An optimal combination of designs and materials to provide durability and biocompatibility to the PIPJ implant has not yet been established [5]. Moreover, the PIPJ implants that have been developed thus far are less durable and are able to achieve less improvement in the functional range of motion than hip or knee joint prostheses [6].

The field of robotic arm applications has recently contributed to the development of implant surfaces using a rolling contact joint (RCJ) mechanism [7, 8]. The RCJ mechanism is defined as a type of restraint system that allows two circular components, connected with flexible straps, to rolling in relation to other contact surface without slip [9]. An implant using the RCJ mechanism offers the advantage of a greater range of motion for the joint, and a lower frictional force that reduces the risk of a chronic inflammatory response to wear debris compared with other implant types [10, 11]. In addition, the RCJ mechanism can help achieve greater stability because of its constrained type design compared to implants using unconstrained designs [12].

Design optimization involves choosing parameters for optimal design from among many alternatives. It requires a cost function that measures the goodness of the design. The cost function is a function of the design parameters and is designed to be minimized when the design is optimal. As per the design optimizations, constraints about the parameters can be needed. We focused on the nonlinear constrained optimization in the present study, because the design optimization used in this study is nonlinear with some constraints. There are many methods to achieve nonlinear constrained optimization, such as Lagrange multiplier, interior-point method, and sequential quadratic programming [13, 14]. The Lagrange multiplier is a method for finding the local minima of a function subject to equality constraints. Both the interior-point method and sequential quadratic programming use the Lagrange multiplier to solve a nonlinear constrained optimization.

The implant design for the upper extremity joints using the RCJ mechanism was first introduced by Bora [15]. However, this design was not tailored to the PIPJ and did not consider the size and kinematics of this joint. Therefore, the present study aimed to propose a novel implant design of the hand PIPJ using an RCJ mechanism and derive an optimal implant design based on the human PIPJ kinematics determined using plain radiographs.

## Materials and methods

### Study participants and acquisition of plain radiographic data

In total, 10 patients (8 female and 2 male) who underwent open reduction and internal fixation for unilateral distal radius fractures, were recruited from December 2018 to March 2019. The inclusion criteria included patients who were aged between 20 and 59 years, who had no history of hand trauma, and who had no history of medication use that could affect bone metabolism. Patients with hand osteoarthritis, including that affecting the PIPJ, or with other upper extremity musculoskeletal conditions were excluded from this study.

We used a true lateral plain radiograph to evaluate the kinematics of the proximal and middle phalanges in relation to their ranges of motion. All images were obtained during surgery after fixation of the distal radius fracture with PIPJ of the second finger positioned at the center of the image. For each participant, 10–12 images were obtained across the PIPJ range of motion from 0 (full extension) to 120° flexion, at intervals of approximately 10°. True lateral plain radiographs of the finger were defined as images in which the two condyles of the proximal phalangeal head overlapped.

Analysis and optimization of data on human PIPJ kinematics and PIPJ implant design using the RCJ mechanism were performed using Matlab® software (version R2018a, Mathworks, Inc., Natick, MA, USA). The NX computer-aided design (CAD) program (version 8.5, Siemens, Munich, Germany) was used to formulate the positions of the proximal and middle phalanges in a two-dimensional (2D) plane based on digitized plain radiographic data.

The study protocol is compliant with the Declaration of Helsinki and was reviewed and approved by the Institutional Review Board of the University Hospital in November 2018 (B-1810/497-002). Written informed consent was obtained from all participants.

### Configuration and parameters of the proposed PIPJ implant

**Fig. 1:**
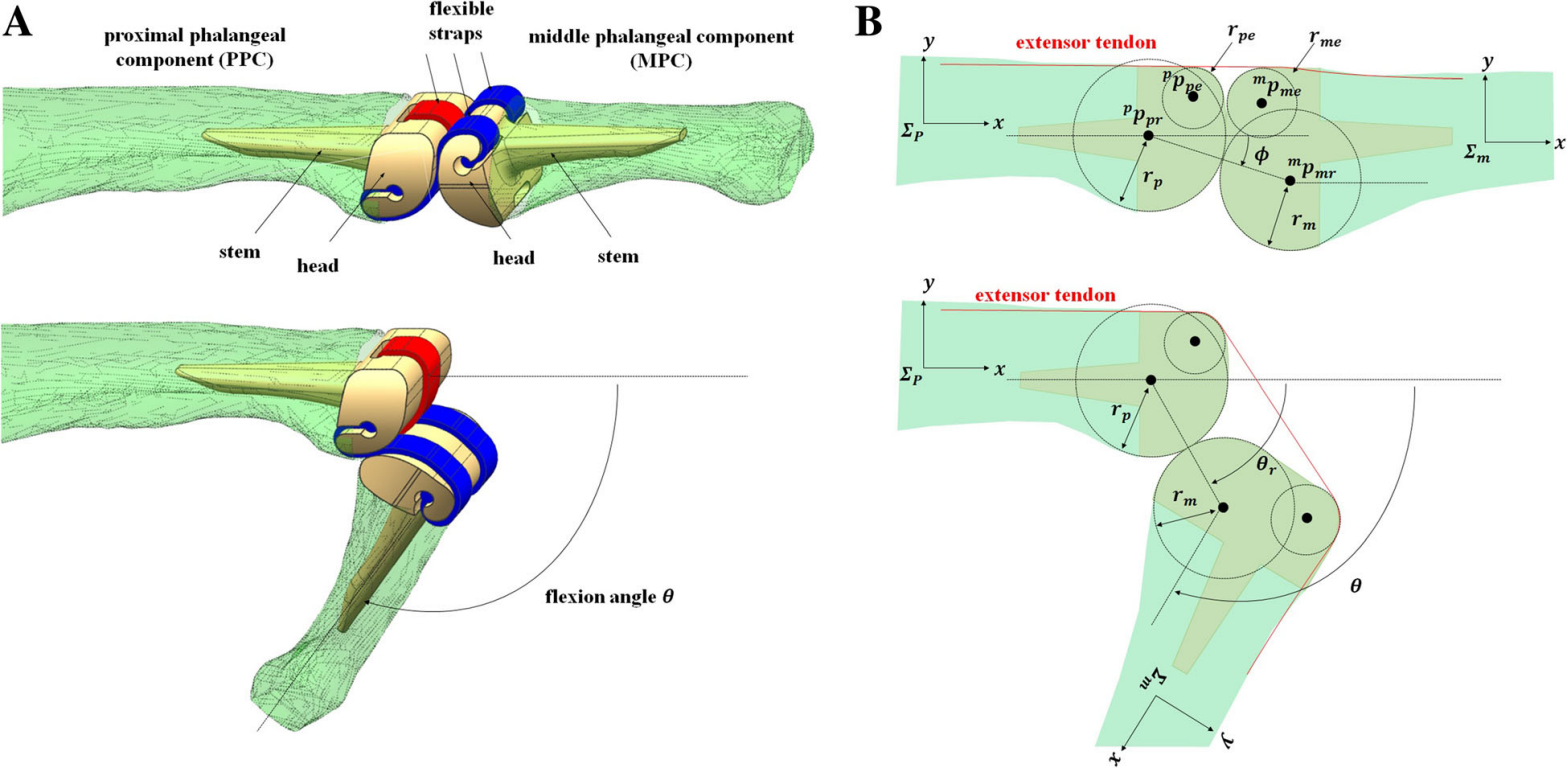
Plain radiograph images and coordinate frames for the middle and proximal phalanges. **a** Three-dimensional model of the proposed proximal interphalangeal joint (PIPJ) in full extension and maximal forward flexion. **b** Kinematic design of the proposed PIPJ implant illustrating the rolling contact surfaces and shapes of the extensor tendon route

Hillberry’s RCJ implant designs, which has been applied to knee joint prostheses, were used [16, 17]. This design allows motion around 1 degree of freedom (1-DOF), comprising PIPJ flexion or extension in the sagittal plane and includes two components: the middle phalangeal component (MPC) and proximal phalangeal component (PPC). Each component has one stem and one head with a circular joint surface; the components are linked by three flexible straps of equal width (Fig. 1a). Among them, two straps are symmetrically located in relation to the third strap, which is located at the center. On the basis of a previous cadaveric study of PIPJ morphology, the width of the radioulnar heads of both components was set a 10 mm and of the straps at 2.5 mm [18].

To determine the mounting positions of PPC and MPC, 2D coordinate frames *Σ*_*p*_ and *Σ*_*m*_ are placed at the proximal and middle phalangeal bones, respectively. The radii of the rolling contact surfaces (RCSs) of the PPC and MPC heads are denoted by *r*_*p*_ and *r*_*m*_, respectively. The positions of the centers of the two circles represented in *Σ*_*p*_ and *Σ*_*m*_ are denoted by ${}^p{p}_p$ and ${}^m{p}_m$, respectively. The two RCSs can be assembled with a nonzero offset angle *ϕ* (Fig. 1b). The shape of the dorsal part of each implant head is important for determining the magnitude of extensor tendon excursion. These two circular surfaces can be defined by their radii *r*_pe_ and *r*_me_ and the center positions ${}^p{p}_{pe}$ and ${}^m{p}_{me}$. The proposed PIPJ implant has 13 design parameters including those related to rolling contact motion (*r*_*p*_, *x*_*p*_, *y*_*p*_, *r*_*m*_, *x*_*m*_, *y*_*m*_, *ϕ*) and extensor excursion (*r*_*pe*_, *x*_*pe*_, *y*_*pe*_, *r*_*me*_, *x*_*me*_, *y*_*me*_), where (*x*_*p*_, *y*_*p*_), (*x*_*m*_, *y*_*m*_), (*x*_*pe*_, *y*_*pe*_), and (*x*_*me*_, *y*_*me*_) represent the components of the position vectors of ${}^p{p}_p$, ${}^p{p}_m$, ${}^p{p}_{pe}$, and ${}^p{p}_{me}$, respectively.

The relationship between two coordinate frames can be expressed by a rotation matrix and a position vector of the origin of a target frame with respect to a base frame. The rotation matrix ${}^p{R}_m$ and the position vector ${}^p{o}_m$ of *Σ*_*m*_ with respect to *Σ*_*p*_ are as follows:

1 $$ {}^p{R}_{\mathrm{m}}=\left[\begin{array}{cc}\cos \left(-\theta \right)& -\sin \left(-\theta \right)\\ {}\sin \left(-\theta \right)& \cos \left(-\theta \right)\end{array}\right],{}^p{o}_m=\left[\begin{array}{c}{x}_o\\ {}{y}_o\end{array}\right] $$

To simplify reporting, the flexion angle *θ* was defined in the clockwise direction and the minus signs at *θ* were applied as shown in Eq. (1). Using this equation, an arbitrary position vector in *Σ*_*m*_ can be transformed to a position vector in *Σ*_*p*_ as follows:

2 $$ {}^pp={}^p{R_m}^mp+{}^p{o}_m $$

where the 2 × 1 vectors ${}^pp={\left[{x}_p,{y}_p\right]}^T$ and ${}^mp={\left[{x}_m,{y}_m\right]}^T$ indicate the same position with respect to the different coordinate frames and each vector consists of two components. For a compact representation, Eq. (2) can be simplified to the homogeneous transformation (HT) matrix as follows:

3 $$ \overset{\sim }{{}^pp}={}^p{T}_m\overset{\sim }{{}^mp}=\left[\begin{array}{cc}& \\ {}{}^p{R}_m& {}^p{o}_m\\ {}& \\ {}0\kern0.75em 0& 1\end{array}\right]\overset{\sim }{{}^mp} $$

where ${}^p{T}_m$ denotes the 3 × 3 HT matrix of *Σ*_*p*_ with respect to *Σ*_*m*_. The notations $\overset{\sim }{{}^pp}={\left[{x}_p,{y}_p,1\right]}^T$ and $\overset{\sim }{{}^mp}={\left[{x}_m,{y}_m,1\right]}^T$ represent 3 × 1 vectors with the same components as ${}^pp$ and ${}^mp$, respectively, and the third unit component.

Using the design parameters, the HT matrix from *Σ*_*p*_ to *Σ*_*m*_ can be calculated. As illustrated in the Fig. 1b, given the flexion angle *θ*, the angle *θ*_r_ from ${}^p{p}_p$ to ${}^p{p}_m$ as follows:

4 $$ {\theta}_r=\phi +\frac{r_m}{r_m+{r}_p}\theta $$

Thus, using *θ* and *θ*_*r*_, the position vector ${}^p{o}_m$ of *Σ*_*m*_ with respect to *Σ*_*p*_ can be rewritten as follows:

5 $$ {}^p{o}_m=\left[\begin{array}{c}{x}_p+\left({r}_p+{r}_m\right)\cos {\theta}_r-{x}_m\cos \theta -{y}_m\sin \theta \\ {}{y}_p-\left({r}_p+{r}_m\right)\sin {\theta}_r+{x}_m\sin \theta -{y}_m\cos \theta \end{array}\right] $$

Therefore, the HT matrix from *Σ*_*p*_ to *Σ*_*m*_ is as follows:

6 $$ {}^p{T}_{m\_ RCJ}\left(\theta \right)=\left[\begin{array}{ccc}\cos \left(-\theta \right)& -\sin \left(-\theta \right)& {x}_p+\left({r}_p+{r}_m\right)\cos {\theta}_r-{x}_m\cos \theta -{y}_m\sin \theta \\ {}-\sin \left(-\theta \right)& \cos \left(-\theta \right)& {y}_p-\left({r}_p+{r}_m\right)\sin {\theta}_r+{x}_m\sin \theta -{y}_m\cos \theta \\ {}0& 0& 1\end{array}\right] $$

This HT matrix indicates the relative motion between the proximal and middle phalangeal bones according to the PIPJ flexion angle when the proposed implant is applied. This relative motion is compared with the human kinematics data to derive the optimal design parameters.

### Determination of the average center of rotation

We hypothesized that the human PIPJ is a hinge joint with a 1-DOF of motion around the center of rotation in the sagittal plane, and we proposed an estimation method that gives the center of rotation of human PIPJ, i.e., average center of rotation (ACR), using plain radiographic data to explain the ideal human PIPJ kinematics.

**Fig. 2:**
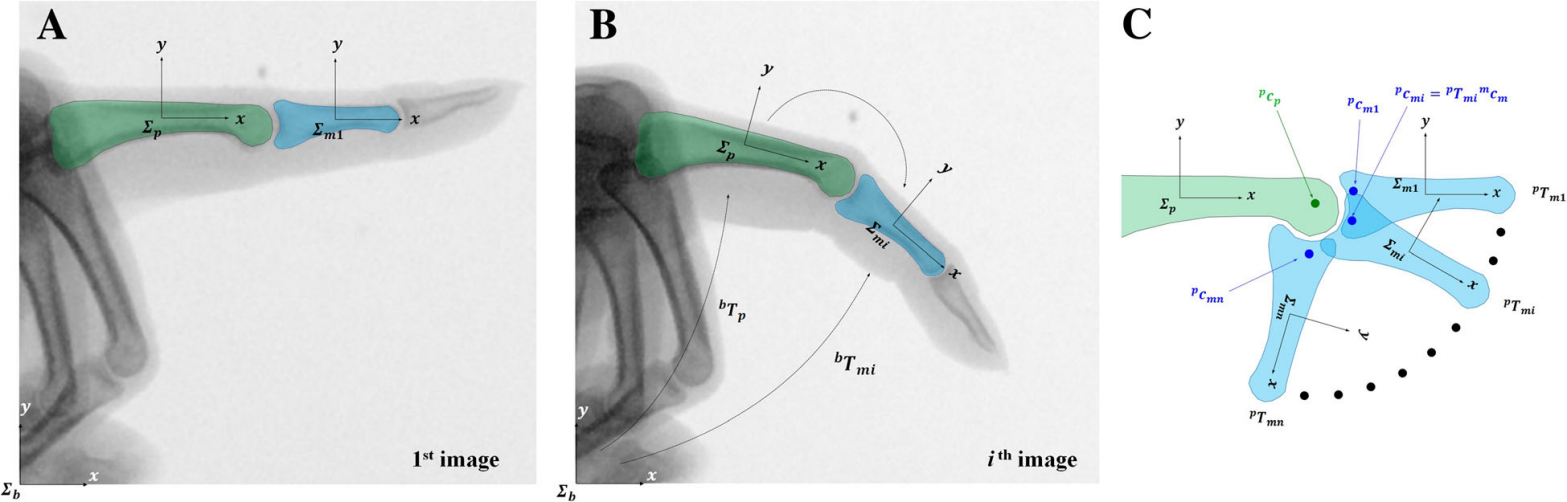
A three-dimensional model of the proximal interphalangeal joint (PIPJ) using the rolling contact joint mechanism. **a** Contours of the proximal (green) and middle (blue) phalangeal bones and the base (*Σ*_*b*_), proximal (*Σ*_*p*_), and middle (*Σ*_*m*1_) coordinate frames in full extension of the PIPJ. **b** The *i*th radiographic image and homogenous transformation (HT) matrices. **c** The average center of rotation (ACR) based on the HT matrices obtained from multiple radiographic images

Using the total number (*n*) of radiographic images, the corresponding *n* number of HT matrices relating the proximal and middle phalanges are calculated. The contours of the proximal and middle phalangeal bones are selected using the CAD program, and the coordinate frames are attached at the centers of the contours (Fig. 2a). The long axis of the proximal and middle phalangeal bones are defined as *x* axes, with the axis perpendicular to the *x* axis defined as *y*. *Σ*_*b*_, *Σ*_*pi*_, and *Σ*_*mi*_ indicate the base frame fixed at an absolute position in the image, the frame of the proximal phalanx of *i*th radiographic image, and the frame of the middle phalanx of *i*th radiographic image, respectively (Fig. 2b); each of these three are set in the same orientation as that of the first image of PIPJ in full extension (Fig. 2a). HTs for the *i*th image, ${}^b{T}_{\mathrm{p}i}$ and ${}^b{T}_{mi}$, are obtained using the CAD program (Fig. 2b). For each image, ${}^p{T}_{mi}$, which represents the relative position and orientation between the proximal and middle phalanges, is calculated using a simple matrix operation as follows:

7 $$ {}^p{T}_{mi}={}^b{T_{pi}^{-1}}^b{T}_{mi} $$

The ACR position is calculated using the acquired *n* numbers of HT matrices assuming that ${}^p{c}_p=\left({x}_{pc},{y}_{pc}\right)$ and ${}^m{c}_m=\left({x}_{mc},{y}_{mc}\right)$ are the ACR positions representing the true center of rotation on the proximal and middle phalanges, respectively. ${}^p{c}_p$ is fixed in *Σ*_*p*_; however, ${}^p{c}_{mi}$, which is the position of ${}^m{c}_m$ with respect to *Σ*_*p*_, varies according to the images. In order for these positions to represent the optimal position of the center of rotation, the following least square distance error should be minimized (Fig. 2c):

8 $$ \underset{c_p,{c}_m}{\mathrm{argmin}}\sum \limits_{i=1}^n{\left\Vert {}^p{c}_{mi}-{}^p{c}_p\right\Vert}^2 $$

The optimal solution for Eq. (8) is achieved using a pseudo-inverse matrix. The error around the *i*th image is expressed using the following HT:

9 $$ {}^p{c}_{mi}-{}^p{c}_p={}^p{T_{mi}}^m{c}_m-{}^p{c}_p=\left[\begin{array}{ccc}\cos {\theta}_i& -\sin {\theta}_i& {x}_{oi}\\ {}\cos {\theta}_i& \cos {\theta}_i& {y}_{oi}\\ {}0& 0& 1\end{array}\right]\left[\begin{array}{c}{x}_{mc}\\ {}{y}_{mc}\\ {}1\end{array}\right]-\left[\begin{array}{c}{x}_{pc}\\ {}{y}_{pc}\\ {}1\end{array}\right] $$

This is arranged to the following desired equation:

10 $$ \left[\begin{array}{cccc}\cos {\theta}_i& -\sin {\theta}_i& 1& 0\\ {}\sin {\theta}_i& \cos {\theta}_i& 0& 1\end{array}\right]\left[\begin{array}{c}{x}_{mc}\\ {}\begin{array}{c}{y}_{mc}\\ {}{x}_{pc}\\ {}{y}_{pc}\end{array}\end{array}\right]-\left[\begin{array}{c}{x}_{oi}\\ {}{y}_{oi}\end{array}\right]\triangleq {A}_iX+{B}_i=0 $$

where *A*_*i*_ and *B*_*i*_ represent a 2 × 4 matrix and a 2 × 1 vector, respectively, and *X* = [*x*_*mc*_ *y*_*mc*_ *x*_*pc*_ *y*_*pc*_ ]^*T*^ is a vector containing the ACR positions for optimizing. By combining Eq. (10) for all *n* of HTs, a resultant equation, for which the error should be minimized, is achieved as follows:

11 $$ \left[\begin{array}{c}{A}_1\\ {}{A}_2\\ {}\vdots \\ {}{A}_n\end{array}\right]X-\left[\begin{array}{c}{B}_1\\ {}{B}_2\\ {}\vdots \\ {}{B}_n\end{array}\right]= AX-B=0 $$

where the sizes of *A* and *B* are 2*n* × 4 and 2*n* × 1 matrices, respectively. Using a pseudo-inverse matrix, the solution that is used to minimize the error of Eq. (8) is obtained as follows:

12 $$ \hat{X}={A}^{+}B={\left({A}^TA\right)}^{-1}{A}^T\ B $$

where *A*^+^ denotes the pseudo-inverse matrix of *A* and, if *A* is full-rank, *A*^+^ is equivalent to (*A*^*T*^*A*)^−1^*A*^*T*^ and the optimal solution $\hat{X}$ contains the ACRs, that is $\hat{X}={\left[{\hat{x}}_{mc}{\hat{y}}_{mc}{\hat{x}}_{pc}{\hat{y}}_{pc}\right]}^T$. The position $\left({\hat{x}}_{pc},{\hat{y}}_{pc}\right)$ best represents the center of rotation with a minimum error when the proximal phalangeal bone is considered as a fixed object; similarly, $\left({\hat{x}}_{mc},{\hat{y}}_{mc}\right)$ is the corresponding position when the middle phalangeal bone is considered as a fixed object. Since the human PIPJ was hypothesized to be a hinge joint, its HT could be obtained based on the ACR derived above, as follows:

13 $$ {}^p{T}_{m\_ Human}\left(\theta \right)=\left[\begin{array}{ccc}\cos \left(-\theta \right)& -\sin \left(-\theta \right)& {\hat{x}}_{pc}-{\hat{x}}_{mc}\cos \left(-\theta \right)+{\hat{y}}_{mc}\sin \left(-\theta \right)\\ {}\sin \left(-\theta \right)& \cos \left(-\theta \right)& {\hat{y}}_{pc}-{\hat{x}}_{mc}\sin \left(-\theta \right)-{\hat{y}}_{mc}\cos \left(-\theta \right)\\ {}0& 0& 1\end{array}\right] $$

### Magnitude of tendon excursions in accordance with the PIPJ flexion angles

The magnitude of extensor tendon excursions was determined in accordance with the PIPJ flexion angles suggested in previous cadaveric studies [19]. The effects of PIPJ motion on the flexor and extensor tendon excursions were evaluated under condition in which the motion of other upper extremity joints was restricted. The excursion of the extensor tendon based on the *extensor digitorum communis* increased by 0.08 mm with each 1° increase in PIPJ forward flexion [19]. Therefore, the magnitude of human PIPJ excursion can be modeled as follows:

14 $$ \Delta {L}_{human}\left(\theta \right)=0.08\ast \theta $$

**Fig. 3:**
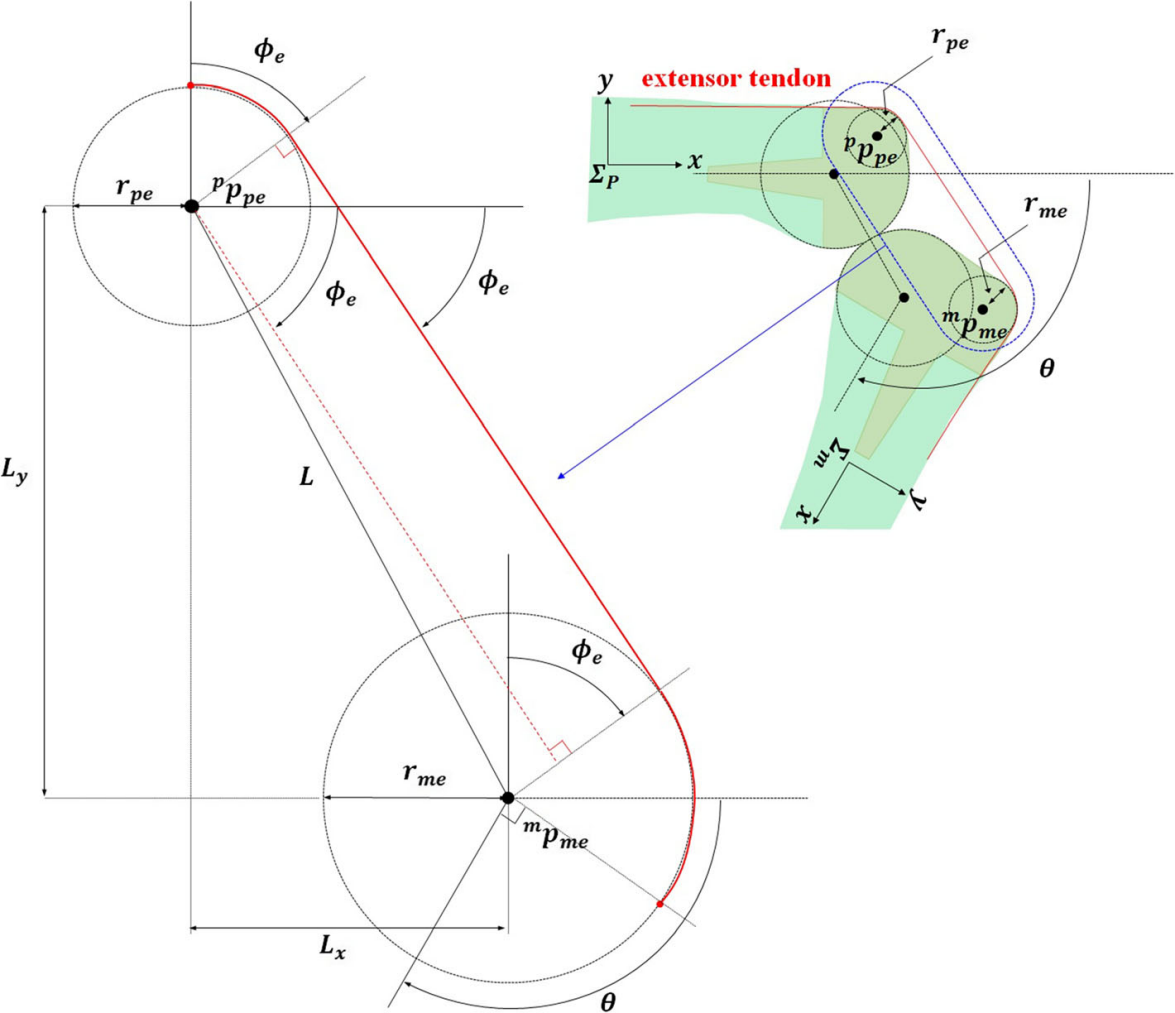
Two circles located on the dorsal side of the proximal interphalangeal joint (PIPJ) implant head. Notations are as follows: *p*_*pe*_ and *p*_*me*_, the centers of the circles in the proximal phalangeal component (PPC) and middle phalangeal component (MPC) heads, respectively; *r*_*pe*_ and *r*_*me*_, the radii of the dorsal circles in the PPC and MPC heads, respectively; *L*, the distance between *p*_*pe*_ and *p*_*me*_; *L*_*x*_ and *L*_*y*_ lengths of *L* projected to the *x* and *y* axes, respectively; *ϕ*_*e*_, the angle in the clockwise direction between the *x* axis and the line tangent to the two circles (red solid line); and *θ*, degrees of PIPJ flexion

The magnitude of extensor tendon excursion according to the flexion angles of the proposed PIPJ implant were determined using the design parameters. As mentioned above, the two circular surfaces on the dorsal part of each component of the implant head affect the magnitude of extensor tendon excursion and are illustrated in Fig. 3. Thus, the length of the extensor tendon with respect to *θ* can be denoted as follows:

15 $$ {L}_{RCJ}\left(\theta \right)={r}_{pe}{\phi}_e+\sqrt{L^2-{\left({r}_{pe}-{r}_{me}\right)}^2}+{r}_{me}\left(\theta -{\phi}_e\right) $$

with $$ {\phi}_e=\mathrm{atan}2\left({L}_y,\kern0.5em {L}_x\right)-\mathrm{asin}\left(\frac{r_{pe}-{r}_{me}}{L}\right) $$,

where *L* is the distance between ${}^p{p}_{pe}$ and ${}^p{p}_{me}$; *L*_*x*_ and *L*_*y*_ are the lengths of *L* projected to the *x* and *y* axes, respectively; *ϕ*_*e*_ is the clockwise angle between the *x* axis and extensor tendon tangential to the two circles; and *L*_*RCJ*_(*θ*) is the length of the extensor tendon of the proposed PIPJ implant. We defined *∆L*_*RCJ*_(*θ*) as the difference between the length of the proposed extensor tendon at the flexion angle *θ* and the length at 0° (full extension), calculated as follows:

16 $$ \Delta {L}_{RCJ}\left(\theta \right)={L}_{RCJ}\left(\theta \right)-{L}_{RCJ}(0) $$

This implies the magnitude of extensor tendon excursion of the proposed PIPJ implant. Therefore, it was essential to properly determine the design parameters so that the function of Eq. (16) could become as consistent as possible with Eq. (14).

### Determination of RCJ design parameters using optimization

We adopted a constrained optimization algorithm using sequential quadratic programming [20] to obtain the optimal RCJ design parameters. Radiographic images were obtained during PIPJ motion from 0 (full extension) to 120° flexion, which reflects the normal range of motion in humans. The proposed PIPJ implant was designed within the same range of motion. The optimization method used in this study comprised one cost function with two components and eight constraints.

The first component of the cost function was designed to allow RCJ to have a similar motion as the human PIPJ. The motions of the human PIPJ and the proposed PIPJ implant were expressed using the HT matrices obtained in Eqs. (6) and (13). The distance error between the human PIPJ and the proposed PIPJ implant motion can be calculated using the position difference between the origin of the two HT matrices. The mean squared error of the distance between the human PIPJ and the proposed PIPJ implant motion is obtained as follows:

17 $$ {C}_p=\frac{1}{n}\sum \limits_{\theta ={0}^{\circ}}^{120^{\circ }}{\left\Vert {}^p{T}_{m_{Human}}\left(\theta \right)\left[\begin{array}{c}0\\ {}0\\ {}1\end{array}\right]-{}^p{T}_{m_{RCJ}}\left(\theta \right)\left[\begin{array}{c}0\\ {}0\\ {}1\end{array}\right]\right\Vert}^2 $$

where the first and second terms in the summation represent the origins of *Σ*_*m*_ in the human PIPJ and the proposed PIPJ implant, respectively. In addition, ‖∙‖^2^ denotes the square of the Euclidean norm of the vector, where the third component of the vector is ignored. The total *n* of errors in the distance from 0 to 120° of the flexion angle *θ* were averaged. In this optimization, the distance error at every 1° was considered; therefore, *n* was set to 121.

The second component of the cost function was designed to minimize the difference between the extensor tendon excursions of the human PIPJ and the proposed PIPJ implant. Using Eqs. (14) and (16), the mean squared error was calculated as follows:

18 $$ {C}_E=\frac{1}{n}\sum \limits_{\theta ={0}^{\circ}}^{120^{\circ }}{\left\Vert \Delta {L}_{human}\left(\theta \right)-\Delta {L}_{RCJ}\left(\theta \right)\right\Vert}^2 $$

Therefore, the overall cost function was defined as

19 $$ C={k}_P{C}_P+{k}_E{C}_E $$

where *k*_*p*_ and *k*_*E*_ are the weighted coefficients of *C*_*p*_ and *C*_*E*_, respectively; these determine the priority and weight between the joint motion and excursion length optimizations. In our optimization, *k*_*p*_ and *k*_*E*_ were given equivalent weightings and set at 1.

In order for all parameters to represent the optimal PIPJ implant using the RCJ mechanism, *C* should be minimized as follows:

20 $$ \underset{r_p,{x}_p,{y}_p,{r}_m,{x}_m,{y}_m,\phi, {r}_{pe},{x}_{pe},{y}_{pe},{r}_{me},{x}_{me},{y}_{me}}{\mathrm{argmin}}C $$

To obtain a feasible RCJ implant design, eight constraints were applied, while minimizing Eq. (20) as follows:

21 $$ \cos {\theta}_d\left({y}_I-{y}_{de}\right)-\sin {\theta}_d\left({x}_I-{x}_{de}\right)\ge 0 $$

22 $$ \cos {\theta}_v\left({y}_V-{y}_{ve}\right)-\sin {\theta}_v\left({x}_V-{x}_{ve}\right)\le 0 $$

*r*_*p*_≥ 5, *r*_*m*_≥ 5 (23)

*r*_*pe*_≥ 2, *r*_*me*_≥ 2 (24)

25 $$ \mid {y}_{pe}\cos {\theta}_d-{x}_{pe}\sin {\theta}_t-{y}_{de}\cos {\theta}_d+{x}_{de}\sin {\theta}_d\mid ={r}_{pe} $$

26 $$ \mid {y}_{pe}\cos {\theta}_d-{x}_{pe}\sin {\theta}_d-{y}_{de}\cos {\theta}_d+{x}_{de}\sin {\theta}_d\mid ={r}_{me} $$

**Fig. 4:**
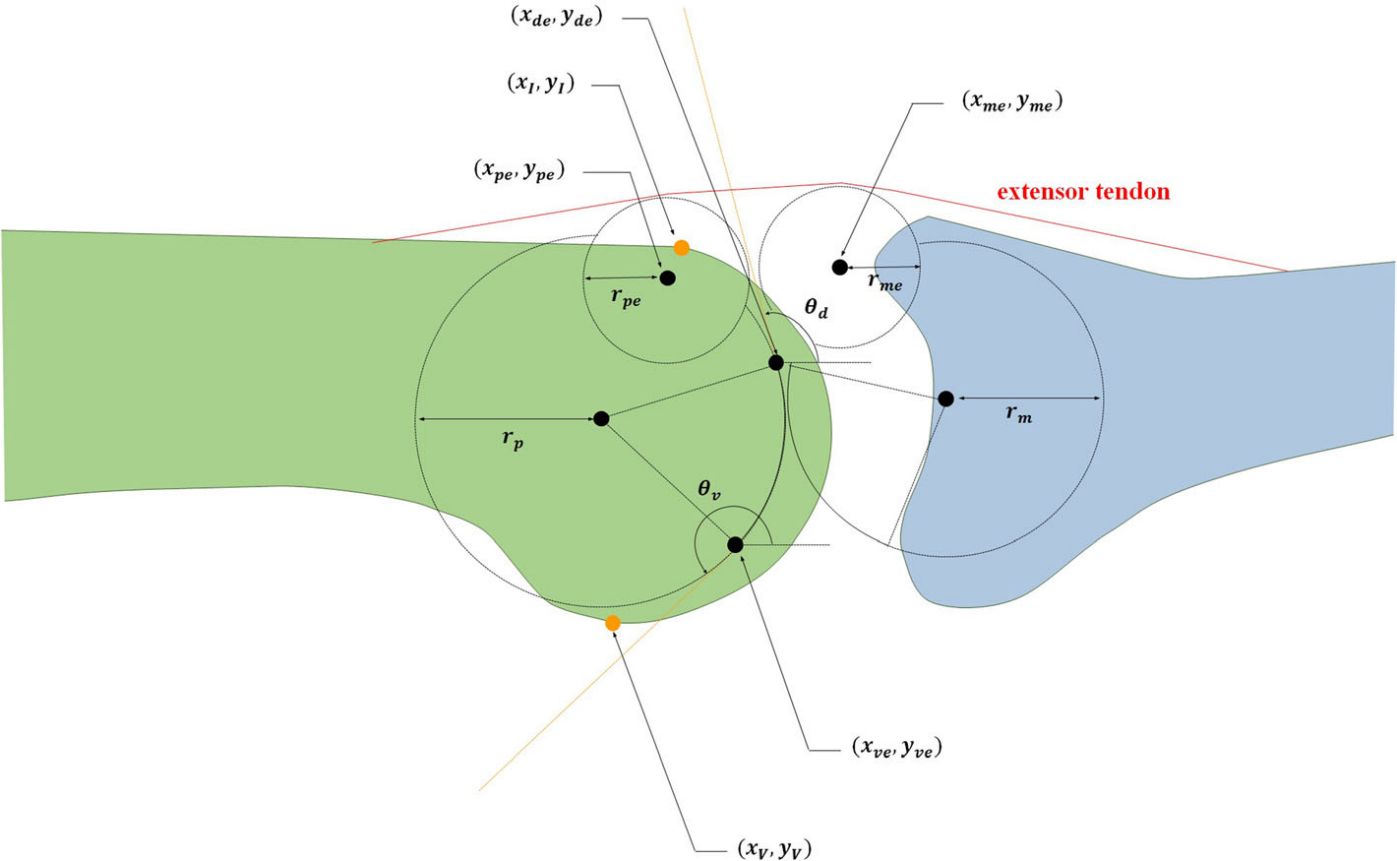
Constraints of the optimization algorithm around the position of the rolling contact surface (RCS). Notations are as follows: *r*_*p*_ and *r*_*m*_, the radii of RCSs in the proximal phalangeal component (PPC) and middle phalangeal component (MPC), respectively; *r*_*pe*_ and *r*_*me*_, the radii of dorsal circles in PPC and MPC heads, respectively; (*x*_*I*_, *y*_*I*_), the position vector of the dorsal infliction point of the proximal phalangeal head; (*x*_*V*_, *y*_*V*_), the position vector of the volar vertex of the proximal phalangeal head; (*x*_*ve*_, *y*_*ve*_), the position vector of the volar end point of RCS; (*x*_*de*_, *y*_*de*_), the position vector of the dorsal end point of RCS. (*x*_*pe*_, *y*_*pe*_) and (*x*_*me*_, *y*_*me*_), the position vectors of the dorsal circles in the PPC and MPC heads, respectively; *θ*_*d*_ and *θ*_*v*_, the angles in counter–clockwise direction between the tangential lines (orange dotted lines) of the dorsal and volar end points of RCS and the *x* axis, respectively

Figure 4 illustrates the positions and angles related to these constraints. The notations (*x*_*de*_, y_*de*_) and (*x*_*ve*_, *y*_*ve*_) represent the positions of the dorsal and volar end points, respectively, on the RCS of PPC; *θ*_*d*_ and *θ*_*v*_ represent the angles of the tangential lines of these dorsal and volar end points in relation to the *x* axis, respectively; and (*x*_*I*_*, y*_*I*_) and (*x*_*V*_*, y*_*V*_) represent the positions of the dorsal infliction point and volar vertex of the proximal phalangeal head, respectively. Equation (21) and (22) place the RCS of PPC at a suitable position for surgery and indicate that the tangential lines of the dorsal and volar end points of the RCS are located distal to the dorsal infliction point (*x*_*I*_*, y*_*I*_) and volar vertex (*x*_*V*_*, y*_*V*_), respectively. On the basis of Eq. (23), the radii of each RCS were set at ≥ 5 mm. These constraints allowed the 2.5-mm flexible strap to endure sufficient contact stress (Hz) [21]. Equation (24) restricts the radii of each circle at the dorsal side of the proposed PIPJ implant hand to ≥ 2 mm in order to prevent the extensor tendon from folding and kinking during flexion. Equations (25) and (26) represent the tangential positions of the dorsal circles of the proposed PIPJ implant head in relation to the dorsal end point (*x*_*de*_, *y*_*de*_).

## Results

### Position of the ACR

**Fig. 5:**
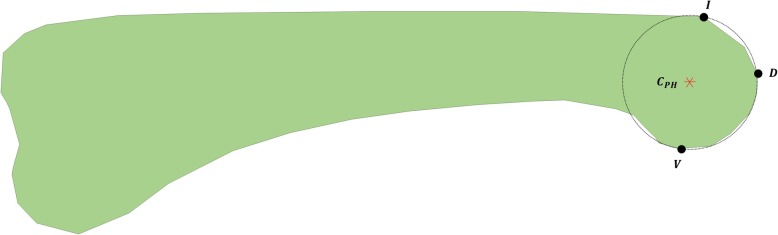
The geometric center of the proximal phalangeal head (*C*_*PH*_) and virtual circle circumscribing three points: the dorsal infliction point (I), volar vertex (V), and distal vertex (D)

**Fig. 6:**
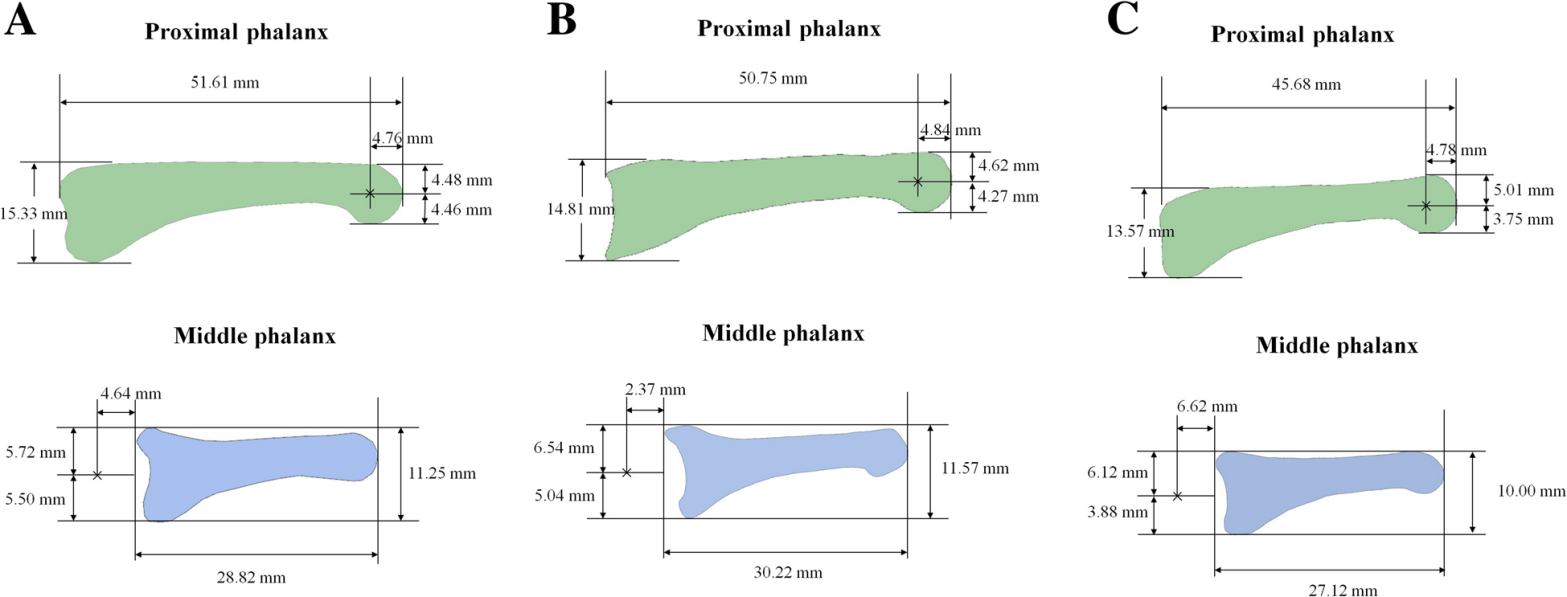
Locations of the average centers of rotation (ACRs) obtained in three participants. The “x” marks appearing in the proximal and middle phalanges represent the ACRs of the respective finger. **a** ACRs of participant 1. **b** ACRs of participant 3. **c** ACRs of participant 8

**Fig. 7:**
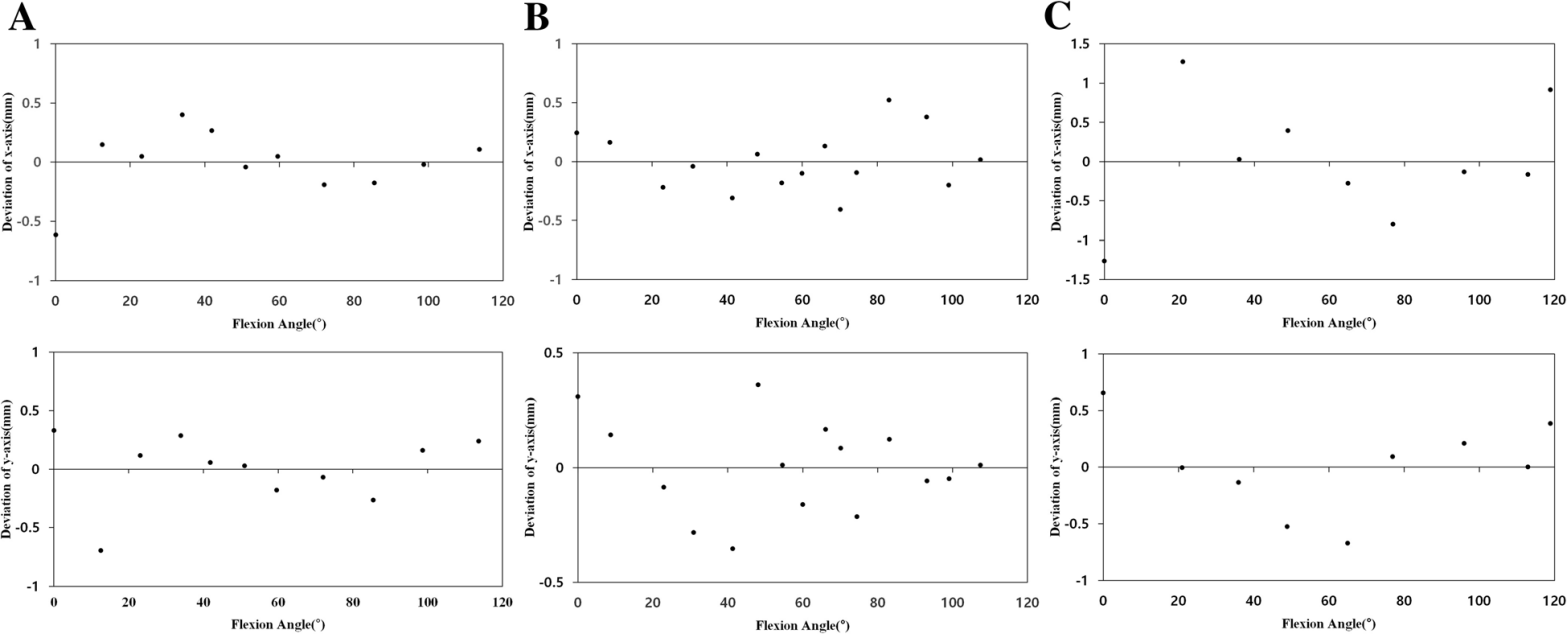
The position of ${}^p{c}_m$ in three participants. ${}^p{c}_m$ of the three participants were located on the *x* and *y* axes centering on ${}^p{c}_p$ in accordance with the proximal interphalangeal joint (PIPJ) range of motion, illustrating the position error between ${}^p{c}_p$ and ${}^p{c}_{mi}.$ **a** Participant 1. **b** Participant 3. **c** Participant 8

**Fig. 8:**
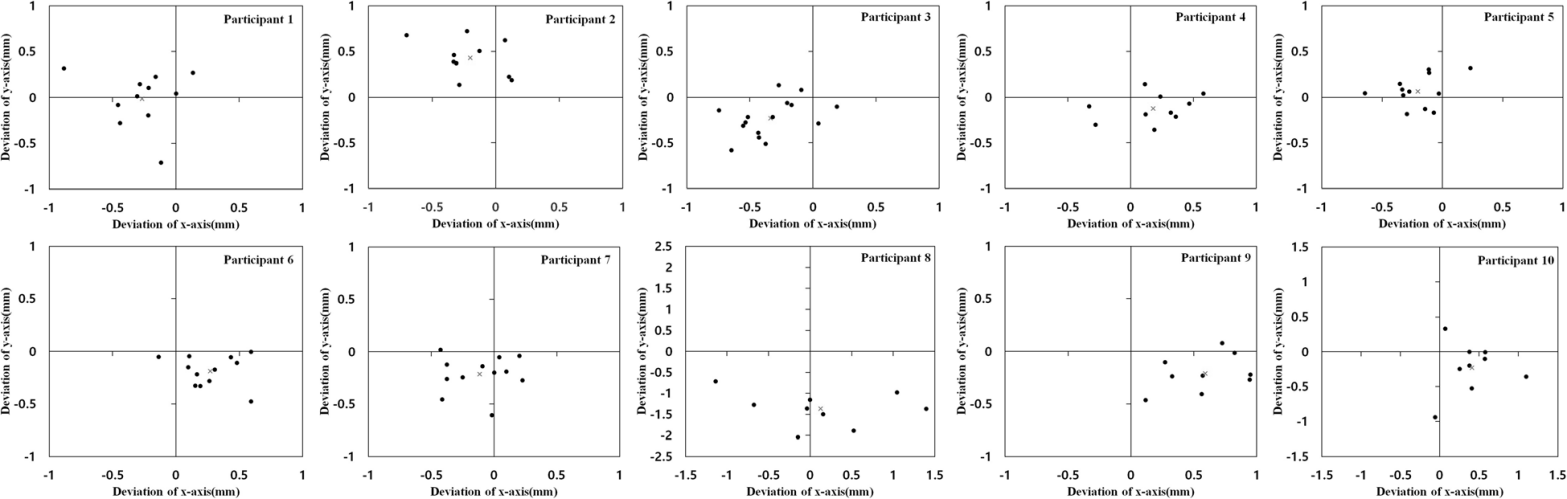
The position of the average centers of rotation (ACRs) in all participants around the geometric center of the proximal phalangeal head (*C*_*PH*_). The black dots and red “x” marks represent the ACRs of the middle and proximal phalanges, respectively. All ACRs tended to be located inferior to the geometric center of the proximal phalangeal head

To represent the standard position of the ACR for each participant, a virtual circle was defined, which circumscribed the triangle connecting the dorsal infliction point, volar vertex, and distal vertex of the proximal phalangeal head. The center of the circle was denoted as *C*_*PH*_ (Fig. 5), and the relative positions of ACRs were expressed on the *x* and *y* axes (Fig. 6). The proximal ACRs were located at a mean of 0.04 ± 0.32 mm (range − 0.34 to 0.59 mm) and − 0.21 ± 0.46 mm (range − 1.37 to 0.43 mm) from *C*_*PH*_ on the *x* and *y* axes, respectively. The position of ${}^m{c}_m$ on the *x* and *y* axes, which was centered on ${}^p{c}_p$ in accordance with the PIPJ range of motion, did not vary with the range of motion and was located within 1.27 mm of ${}^p{c}_p$ (Fig. 7). In addition, the proximal ACRs for each participant tended to be positioned inferior to *C*_*PH*_ (Fig. 8).

### Determination of the RCJ design parameters

The mean values of *r*_*p*_, *r*_*m*_, *r*_*pe*_, and *r*_*me*_ determined using the constrained optimization algorithm were 11.49 ± 1.53 mm (range 8.25–13.27 mm), 5.00 ± 0.00 mm, 2.15 ± 0.28 mm (range 2.00–2.87 mm), and 2.03 ± 0.09 mm (range 2.00–2.30 mm), respectively. Table 1 shows the long–axis length of the proximal and middle phalanges, anteroposterior diameters of the proximal phalangeal head and middle phalangeal base, and values of parameters determined for each participant.

**Table 1:** Design parameters of proposed proximal interphalangeal joint (PIPJ) implant

	Length of long axis (proximal phalanx, middle phalanx) (mm)	Dorso-volar diameter (proximal phalangeal head, middle phalangeal base) (mm)	*r*_*p*_ (mm)	*r*_*m*_ (mm)	*r*_*pe*_ (mm)	*r*_*me*_ (mm)	Components of position vector: ^*p*^*p*_*p*_ = (*x*_*p*_, *y*_*p*_)	Components of position vector: ^*p*^*p*_*m*_ = (*x*_*m*_, *y*_*m*_)	Components of position vector: ^*p*^*p*_*pe*_ = (*x*_*pe*_, *y*_*pe*_)	Components of position vector: ^*p*^*p*_*me*_ = (*x*_*me*_, *y*_*me*_)	Offset angle: *ϕ* (°)
Participant 1	(51.61, 28.82)	(8.94, 11.25)	12.77	5.00	2.00	2.00	(− 11.58, 1.87)	(6.11, 3.45)	(− 0.86, 2.83)	(3.13, 3.18)	− 5.09
Participant 2	(46.98, 28.49)	(9.25, 12.17)	12.85	5.00	2.00	2.00	(− 11.95, 0.31)	(5.48, 4.18)	(− 1.36, 2.66)	(2.55, 3.53)	− 12.51
Participant 3	(50.73, 30.22)	(8.89, 11.57)	13.27	5.00	2.00	2.00	(− 11.63, 2.02)	(6.61, 3.04)	(− 0.38, 2.65)	(3.62, 2.87)	− 3.21
Participant 4	(34.04, 18.93)	(6.17, 7.70)	8.25	5.00	2.87	2.30	(− 7.39, 1.83)	(5.77, 3.34)	(− 2.05, 2.44)	(3.10, 3.03)	− 6.54
Participant 5	(41.46, 24.03)	(7.27, 8.99)	10.78	5.00	2.11	2.00	(− 9.78, 1.28)	(5.81, 3.76)	(− 1.21, 2.65)	(2.84, 3.29)	− 9.05
Participant 6	(40.89, 23.84)	(7.77, 9.37)	12.05	5.00	2.00	2.00	(− 10.49, 2.71)	(6.56, 2.89)	(− 0.44, 2.81)	(3.56, 2.85)	− 0.58
Participant 7	(42.07, 24.32)	(7.30, 8.98)	10.11	5.00	2.38	2.00	(− 9.21, 0.98)	(5.62, 3.89)	(− 1.62, 2.47)	(2.68, 3.31)	− 11.13
Participant 8	(45.67, 27.12)	(8.76, 10.00)	12.46	5.00	2.00	2.00	(− 10.83, 2.15)	(6.62, 2.94)	(− 0.38, 2.62)	(3.62, 2.80)	− 2.56
Participant 9	(38.31, 22.51)	(7.52, 8.44)	11.24	5.00	2.00	2.00	(− 10.04, 1.10)	(5.99, 3.67)	(− 0.92, 2.56)	(3.03, 3.20)	− 9.10
Participant 10	(41.57, 22.26)	(7.85, 9.44)	11.08	5.00	2.10	2.00	(− 10.19, 1.15)	(5.66, 3.87)	(− 1.34, 2.67)	(2.70, 3.36)	− 9.75

*r*_*p*_ radii of rolling surface of the heads of proximal phalangeal component, *r*_*m*_ radii of rolling surface of the heads of middle phalangeal component, *r*_*pe*_ radii of circular surface located at the dorsal part of proximal phalangeal component head, *r*_*me*_ radii of circular surface located at the dorsal part of middle phalangeal component head

The mean value of the position errors between the centers of rotation of the PIPJ implant derived from the optimized algorithm and ACRs of the human PIPJ determined using plain radiographs was 1.25 ± 0.32 mm (range 0.63–1.81 mm), and the mean difference between the extensor tendon excursions of the proposed PIPJ implant and those of the human PIPJ was 0.16 ± 0.04 mm (range 0.1–0.24 mm). Both mean values were obtained for each of the 10 participants and were calculated as the mean of values measured from 0 (full extension) to 120° flexion in 1° increments. Therefore, the proposed PIPJ implant exhibited an acceptable kinematic range compared with that of the human PIPJ.

**Fig. 9:**
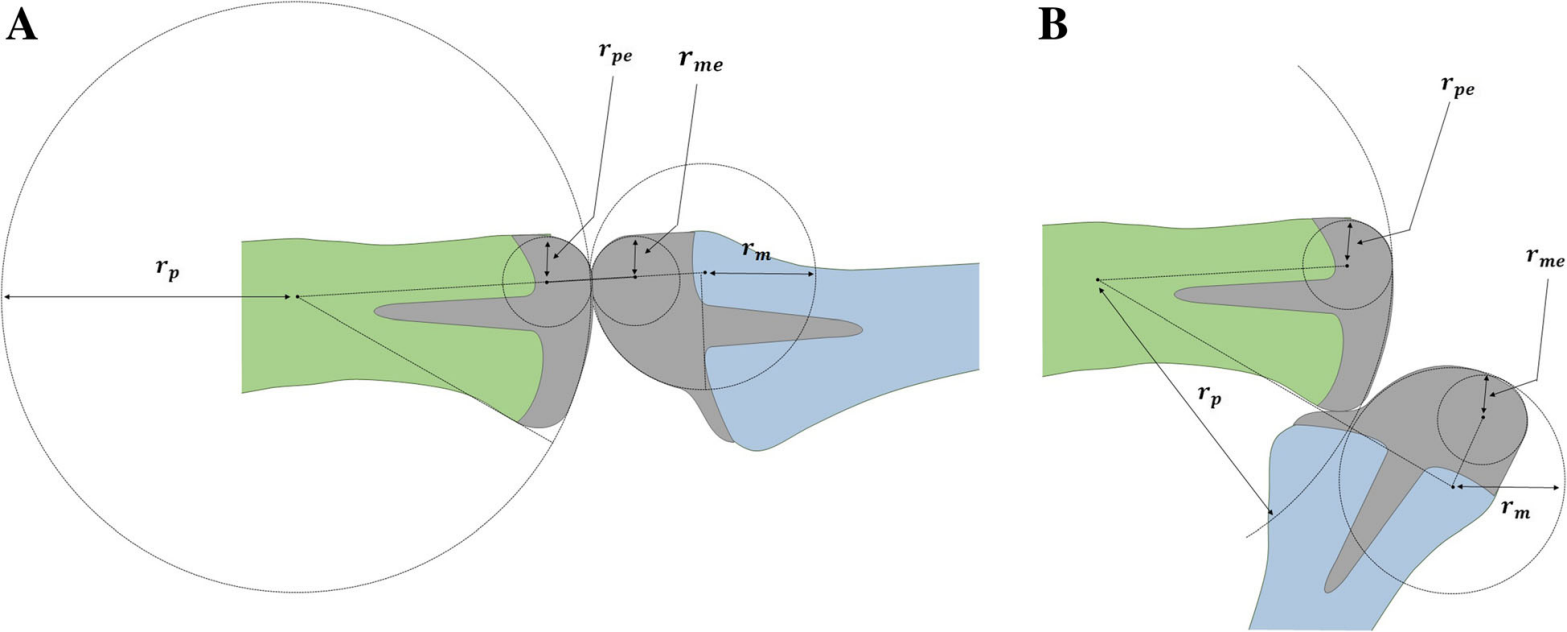
The final optimized design of proposed proximal interphalangeal joint (PIPJ) implant located on the phalangeal bone. **a** The position at 0° (full extension) of the PIPJ. **b** The position at 120° flexion of the PIPJ. Notations are as follows: *r*_*p*_ and *r*_*m*_, the radii of rolling contact surfaces (RCSs) in the proximal phalangeal component (PPC) and middle phalangeal component (MPC), respectively, and *r*_*pe*_ and *r*_*me*_, the radii of dorsal circle in the PPC and MPC heads, respectively

**Fig. 10:**
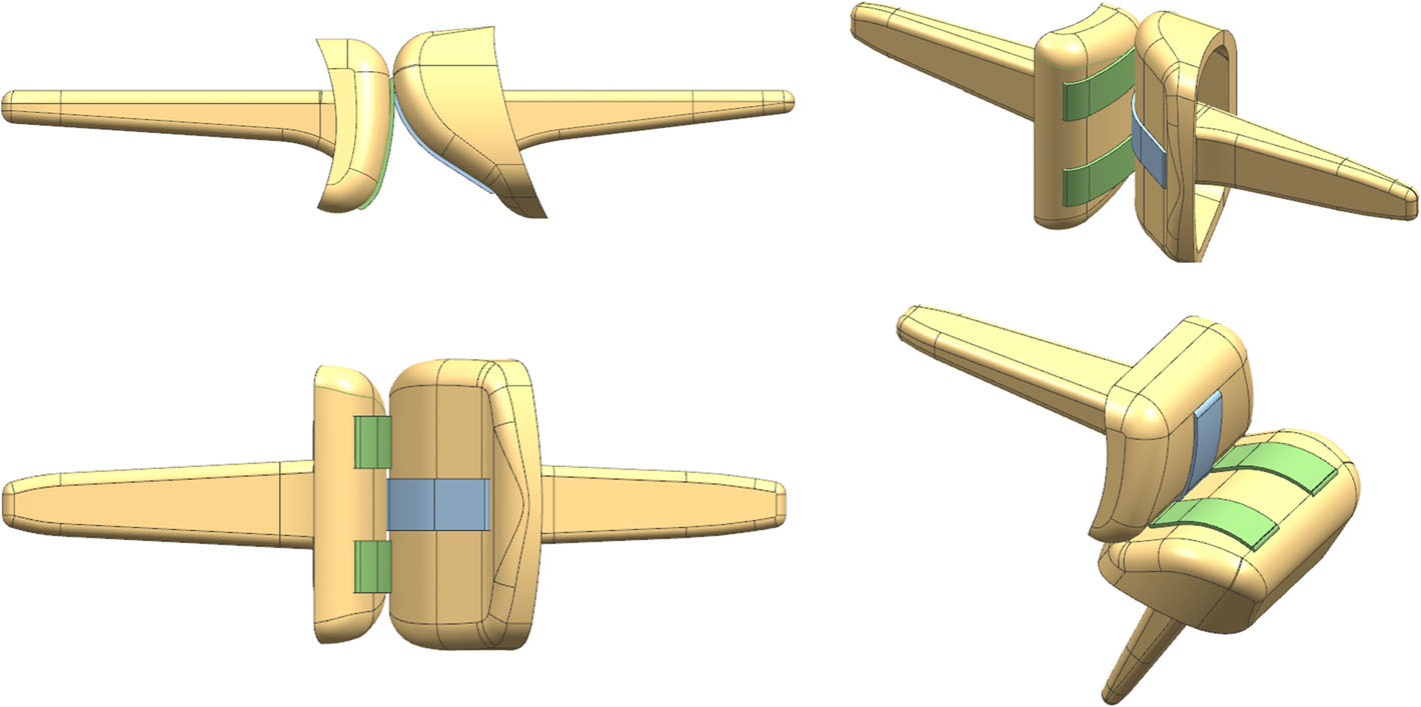
A three-dimensional (3D) model of the optimized proximal interphalangeal joint (PIPJ) implant

Using the determined parameters, the proposed PIPJ implant was designed using Matlab® (Fig. 9); a concordant three-dimensional (3D) model was created using the CAD program (Fig. 10).

## Discussion

The most essential criterion for developing a PIPJ implant using the RCJ mechanism is to achieve a joint design that precisely simulates human biomechanical properties. The RCJ motion should be similar to the human PIPJ kinematics, and the extensor and flexor tendon excursion should be within acceptable magnitudes as compared to those of the human PIPJ [22]. Accurate motion analysis is essential for reducing the human PIPJ kinematics. The initial step of our analysis involved determination of the PIPJ center of rotation.

Various methods to determine the center of rotation have been developed, which are based on calculating the finite displacement of a rigid body [23, 24]; however, this finite center of rotation becomes inaccurate if the displacement approaches zero. Thus, iterative numerical optimizations can be used to achieve the true center of rotation [25]. ACR proposed in this study provides a simple and efficient closed-form solution of ACR in the sagittal plane using data from multiple radiographic images to minimize the distance error from the optimal center of rotation.

In addition to the flexion–extension in the sagittal plane, PIPJ kinematics includes abduction–adduction, axial rotation, and sagittal rotation [26]. However, the proposed PIPJ implant was based on Hillberry’s RCJ design, which reproduced only 1-DOF of motion, namely flexion–extension, in sagittal plane. Abduction–adduction or rotational motion represents a small proportion of the total PIPJ motion [25], and flexion–extension in the sagittal plane better reflects the motions performed in daily life [27]. Among the PIPJ implant designs reported to date, a silicone implant with a volar approach has been found to exhibit the best durability and least complications [28]; this implant was a 1-DOF hinge type joint allowing flexion–extension in the sagittal plane, consistent with our proposed PIPJ implant using an RCJ mechanism. Recently, surface replacement arthroplasties with ≥ 2-DOF have shown poorer clinical outcomes than silicone implants [28]. Thus, our PIPJ implant with 1-DOF has a suitable design and can reproduce human PIPJ kinematics.

The present study proposed a constrained type of PIPJ implant with 1-DOF characterized by a hinge joint. Previous cadaveric motion analysis study has reported that the displacement of the center of rotation in the sagittal plane through the PIPJ range of motion was ≤ 0.30 mm [25]. ACRs in this study showed position errors within 1.27 mm, which were not correlated with the PIPJ flexion angle at a relatively constant position (Fig. **7**). Therefore, the proposed PIPJ implant with a 1-DOF hinge joint positioned on ACR appears to be appropriate.

A constrained optimization algorithm was applied to determine the design parameters of the proposed PIPJ implant. The proximal position of the RCJ surface, in relation to the neck of the proximal phalanx, was detected without including the condition for the RCJ surface position in the optimization algorithm. A joint rotation occurs on the RCJ surface in implants using an RCJ mechanism [17]; therefore, the RCJ surface of the proposed PIPJ implant was optimized to a position close to the center of rotation derived from human PIPJ kinematics. However, removal of a substantial amount of proximal phalanx bone stock is necessary during surgery to achieve the proximal position of the RCJ surface proximal to the neck of the proximal phalanx. In addition, PIPJ could become unstable as a result of injury to the collateral ligament during implantation [26]. Therefore, we included a condition in the constrained optimization algorithm requiring the tangential line extending from the dorsal and volar sides of the RCJ surface to be positioned distal to the dorsal infliction point and volar vertex of the proximal phalangeal head. Consequently, the optimized RCJ surface was located inside the head of proximal phalanx, and it could preserve a considerable amount of bone stock from the proximal phalangeal head and avoid damage to the collateral ligament during surgery, thereby contributing to improved postoperative PIPJ stability.

The interior-point method is an effect and rapid method for a large number of parameters and constraints. However, its solution can be relatively less accurate than those of other algorithms because the internal process of the algorithm keeps iterates away from inequality constraint boundaries. The sequential quadratic programming is appropriate for small- or medium-scale optimization; however, it is relatively slow during the optimization of a large number of parameters and constraints. In this study, there were 13 parameters and eight constraints, and the sequential quadratic programming was adopted to solve the optimization.

The optimized results demonstrated that the diameter of the RCS of MPC was 5 mm and that of PPC was 8–13 mm; these findings can be explained by two factors. First, because the human PIPJ is hinged around the center of rotation, the optimized RCS diameter should be minimized in order to mimic human kinematics. However, to adequately endure the contact stress, the RCS diameter should be set at ≥ 5 mm; therefore, the optimized diameters of MPC and PPC should approach this value. Second, ACR determined using plain radiographic data was located near the neck of the proximal phalanx. As the RCJ center of rotation was located on RCS, a larger PPC diameter was required to enable RCS to be located near the neck of the proximal phalanx, thus, resembling human PIPJ kinematics.

Decreased length of the phalangeal bone may contribute to relative extensor tendon elongation. A previous cadaveric study has suggested that a decrease of 1 mm could lead to a 12° extension lag [29], that could cause functional limitation of the hand. In this study, equal weightings were given to the cost function components of the extensor tendon and the center of rotation to reduce the potential for bone–tendon length discrepancy following PIPJ implant insertion; thus, optimization was performed to maintain the long–axis phalangeal bone length. The final implant design demonstrated that the proposed PIPJ implant was on average 1.1 mm shorter than the sum of the lengths of the normal proximal and middle phalanges. However, given the compensation provided by the extensor tendon excursion reserve effect and the intrinsic muscle function [30], the proposed PIPJ implant design could show a few degrees of extension lag on PIPJ that does not significantly affect the hand function.

This study has certain limitations. First, because we used 2D plain radiographs, 3D PIPJ motions such as abduction–adduction or rotation could not be evaluated. Second, although the size and position of the proposed PIPJ implant were designed to reproduce human PIPJ kinematics and anatomy, the various technical problems that might occur during total PIPJ replacement arthroplasty cannot be ruled out. Third, because the 3D structures were projected in a 2D plane, the original size and contour of the PIPJ structures could be distorted. Fourth, because the PIPJ range of motion and magnitude of extensor excursion may be dependent to each other, it may cause the error in the cost function. However, the errors calculated from the two cost functions were 1.25 ± 0.32 mm and 0.16 ± 0.04 mm, respectively; these were small enough to be clinically significant. Therefore, the feasibility of this study is maintained.

## Conclusions

To our knowledge, this is the first study to design a PIPJ implant using the RCJ mechanism to fit the human PIPJ structure. Further, the concept of ACR was newly introduced to determine the center of rotation during the human PIPJ range of motion. A novel PIPJ implant design using the RCJ mechanism demonstrated acceptable features in terms of tendon excursions and human PIPJ kinematics. Future studies on the biomechanical properties of implant materials and strap applications may help accelerate the clinical application of the RCJ mechanism implants in total PIPJ arthroplasty.

## Data Availability

The datasets used and analyzed during the current study are available from the corresponding author on reasonable request.

## Abbreviations

1-DOF: 1 degree of freedom
2D: Two-dimensional
2-DOF: 2 degrees of freedom
3D: Three-dimensional
ACR: Average center of rotation
CAD: Computer-aided design
HT: Homogenous transform
MPC: Middle phalangeal component
PIPJ: Proximal interphalangeal joint
PPC: Proximal phalangeal component
RCJ: Rolling contact joint
RCS: Rolling contact surface
